# Case Report: PD-1/CTLA-4 dual checkpoint blockade(QL1706) in advanced pulmonary squamous cell carcinoma complicated by multidrug-resistant tuberculosis after multiple lines of immunotherapy

**DOI:** 10.3389/fonc.2026.1775568

**Published:** 2026-04-16

**Authors:** Li Huang, Hao Li

**Affiliations:** 1 Department of Oncology, Guiyang Public Health Clinical Center, Guiyang, China; 2 Department of Oncology, Guiyang Cancer Hospital, Guiyang, China; 3 Department of Oncology, Guiyang Fifth People’s Hospital, Guiyang, Guizhou, China

**Keywords:** immunotherapy rechallenge, multidrug-resistant tuberculosis, non-small cell lung cancer, PD-1/CTLA-4, QL1706, squamous cell carcinoma

## Abstract

Immune checkpoint inhibitors (ICIs) are standard care for advanced non-small cell lung cancer (NSCLC), yet patients with active tuberculosis (TB) are consistently excluded from clinical trials. Furthermore, data guiding the use of dual PD-1/CTLA-4 blockade in the presence of multidrug-resistant TB (MDR-TB) is lacking. We report a 56-year-old man with stage IVA pulmonary squamous cell carcinoma (SqNSCLC) who progressed on multiple lines of therapy, including chemotherapy plus pembrolizumab, docetaxel, and the PD-1/VEGF bispecific antibody ivonescimab. During treatment, he developed secondary MDR-TB. Following stabilization with a WHO-recommended all-oral MDR-TB regimen and palliative radiotherapy for a progressive metastatic lesion, he was rechallenged with QL1706 (a unified PD-1/CTLA-4 bispecific antibody). The patient achieved sustained stable disease with a progression-free survival (PFS) of approximately 11 months. Crucially, strict multidisciplinary monitoring confirmed no TB reactivation or dissemination, and no grade ≥3 immune-related adverse events occurred. This case provides the first clinical evidence that, in carefully selected patients with controlled MDR-TB, dual checkpoint blockade with QL1706 is a feasible salvage strategy, provided that TB is rigorously managed.

## 1 Introduction

The intersection of advanced lung cancer and tuberculosis (TB) presents a formidable clinical dilemma. While immune checkpoint inhibitors (ICIs) targeting PD-1 and CTLA-4 have transformed lung cancer survival (1), the PD-1 pathway is physiologically essential for containing Mycobacterium tuberculosis (Mtb) within granulomas. Therapeutic blockade of this pathway can disrupt granuloma integrity, leading to the “ICI-related TB paradox” and potentially fatal reactivation (2, 3). Consequently, patients with active TB are universally excluded from ICI trials, leaving a knowledge gap regarding optimal management—particularly concerning multidrug-resistant TB (MDR-TB). While dual immunotherapy (CTLA-4 plus PD-1 blockade) offers enhanced antitumor efficacy, it is generally associated with higher rates of immune-related adverse events (irAEs) than PD-1 blockade alone.

QL1706 (iparomlimab/tuvonralimab) is a novel bifunctional bispecific antibody capable of simultaneous PD-1 and CTLA-4 blockade. Engineered on the MabPair^®^ platform, it is designed to maintain synergistic antitumor efficacy while reducing the systemic toxicity typically associated with anti-CTLA-4 antibodies (4–6). QL1706 has shown promising activity in ICI-experienced NSCLC (5, 7), yet its safety in the context of chronic infection remains unknown. Here, we report a patient with advanced SqNSCLC and concomitant MDR-TB who successfully underwent rechallenge with QL1706 following effective anti-TB therapy, achieving durable disease control without TB reactivation.

## 2 Case presentation

### 2.1 Oncologic history and MDR-TB emergence

**Figure 1:**
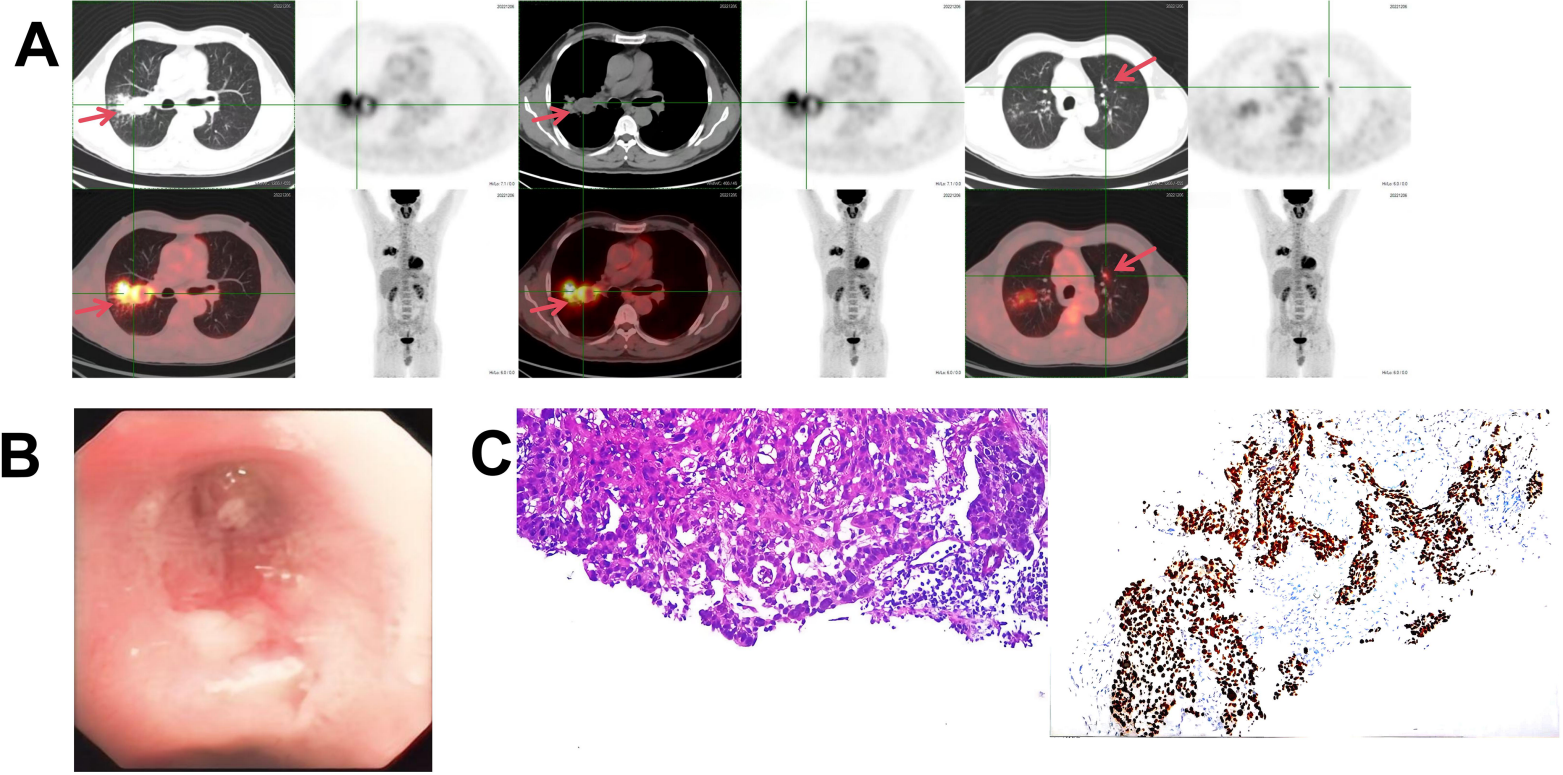
**(A)** ^18^F-FDG PET-CT showing a hypermetabolic cavitary mass in the right upper lobe (primary tumor) and a metastatic nodule in the left upper lobe. **(B)** Bronchoscopy revealing neoplastic occlusion of the right upper lobe bronchus. **(C)** Histopathology confirming squamous cell carcinoma (Left: H&E staining; Right: diffuse nuclear positivity for p40).

A 56-year-old man presented in late 2022 with stage IVA squamous cell carcinoma of the lung (cT2bN1M1b), confirmed by PET-CT and histopathology via bronchoscopy (**Figure 1**). Between May 2023 and June 2024, he progressed through multiple lines of systemic therapy, including first-line pembrolizumab plus chemotherapy, second-line docetaxel, and third-line ivonescimab (PD-1/VEGF bispecific antibody). However, during third-line therapy, the patient developed recurrent fever and worsening pulmonary infiltrates. Sputum analysis confirmed secondary MDR-TB involving both lungs, with resistance to isoniazid, rifampicin, ethambutol, and fluoroquinolones. Immunotherapy was immediately suspended.

### 2.2 MDR-TB management and bridge therapy

An all-oral MDR-TB regimen comprising bedaquiline, linezolid, clofazimine, and cycloserine was initiated in accordance with WHO guidelines (8). Over two months, fever and hemoptysis resolved, and inflammatory infiltrates regressed. While TB was stabilizing, a metastatic lesion in the left upper lobe progressed. To control tumor burden without immediate systemic immunosuppression, palliative intensity-modulated radiotherapy (IMRT, 66 Gy/33f) was administered, resulting in marked shrinkage of the metastatic lesion. By December 2024, repeated sputum cultures converted to negative, creating a window for systemic therapy rechallenge.

### 2.3 Immunotherapy rechallenge with QL1706

Given the prior failure of PD-1 monotherapy and PD-1/VEGF blockade, the multidisciplinary team opted for a more potent immunomodulatory strategy using QL1706 to leverage the synergistic effect of CTLA-4 blockade. The patient commenced QL1706 (250 mg IV q3w) in January 2025. The treatment course included an initial phase of monotherapy, a brief combination phase with chemotherapy (nab-paclitaxel/gemcitabine, discontinued due to Grade 2 neuropathy and thrombocytopenia), and a subsequent maintenance phase with QL1706 monotherapy.

### 2.4 Clinical outcome and safety

**Figure 2:**
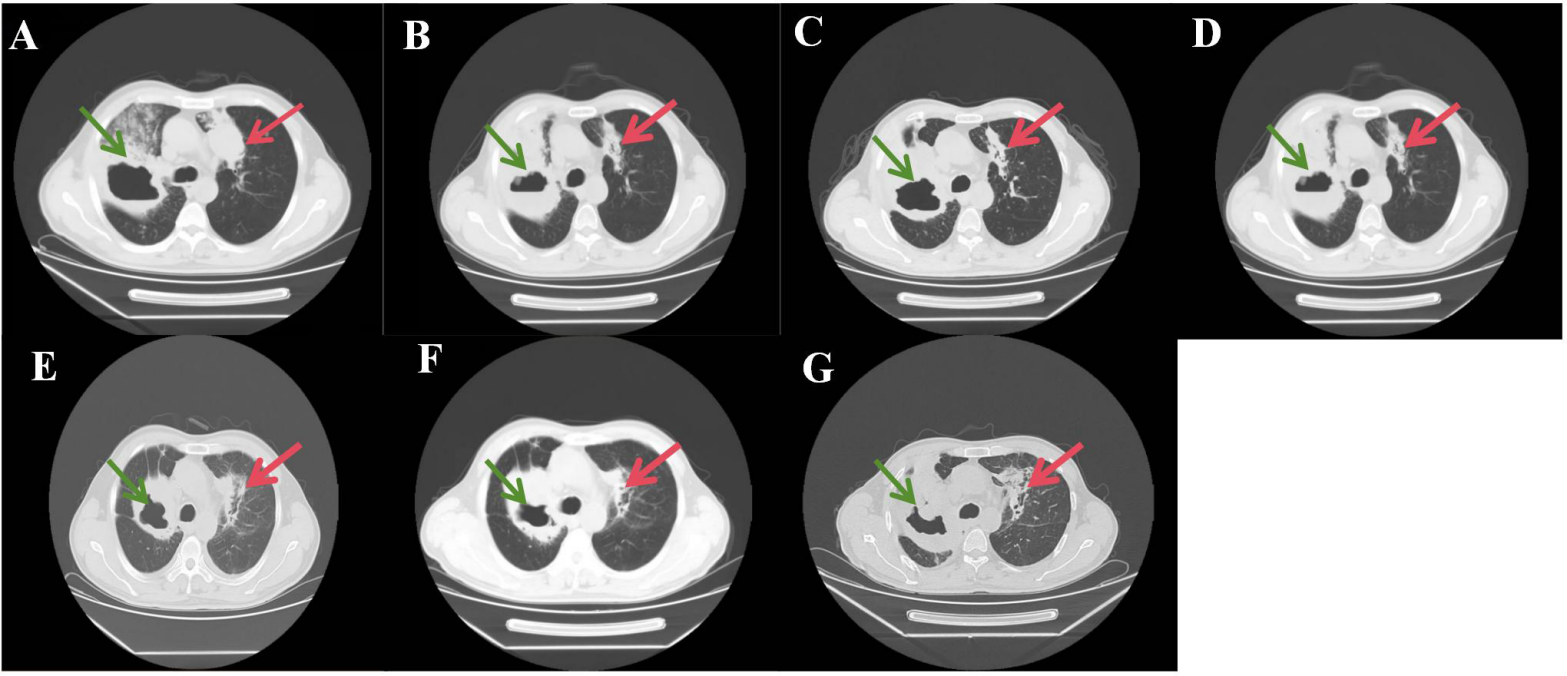
**(A–G)** track the primary right upper lobe cavitary lesion from pre-treatment baseline through 11 months of follow-up. Despite a transient radiographic flare in June 2025 **(E)** attributed to peritumoral inflammation, the solid tumor component remained overall stable consistent with Stable Disease (SD), and no new lesions appeared.

**Figure 3:**
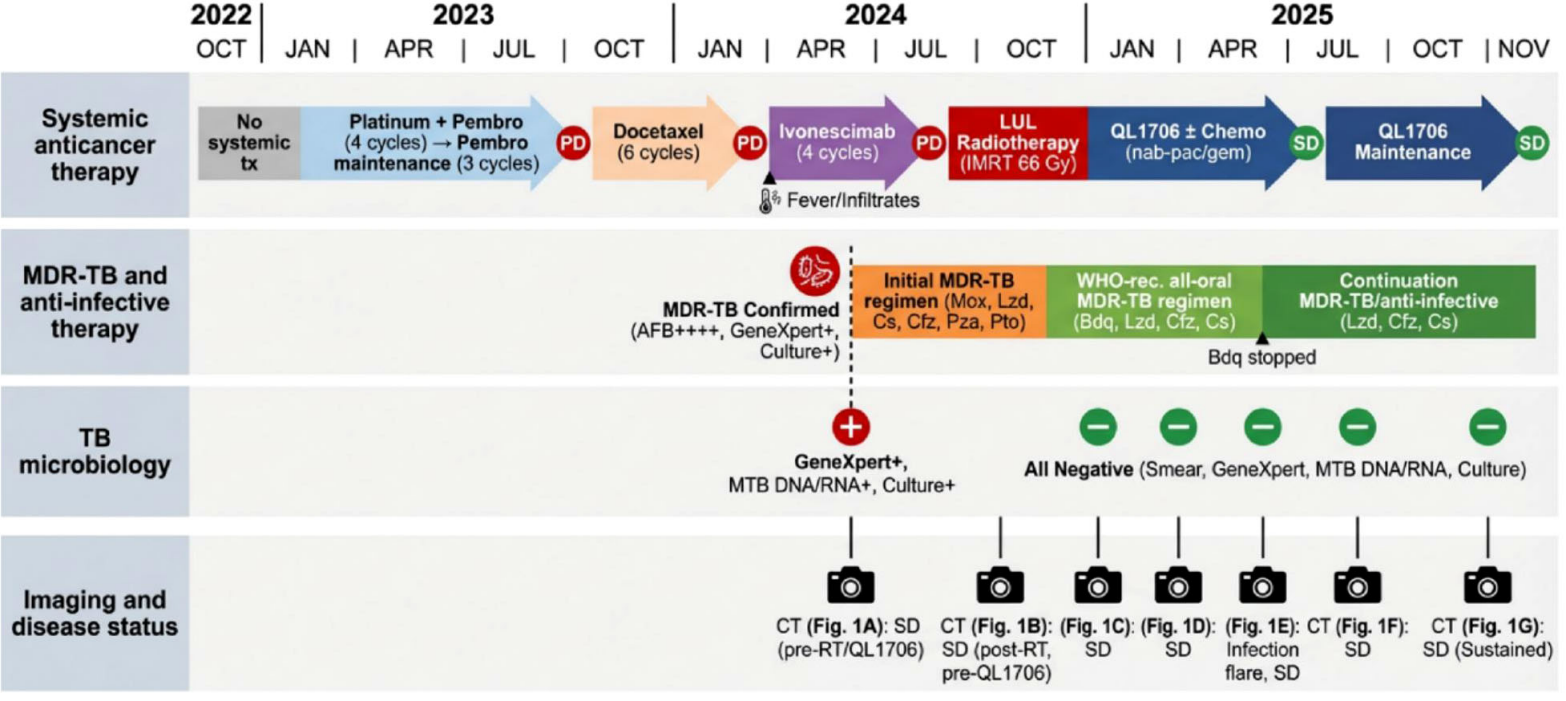
Clinical timeline. Schematic illustration of the patient’s treatment course, detailing the sequence of systemic therapies, the interval of MDR-TB management, and the timing of QL1706 rechallenge relative to microbiologic control.

Serial imaging demonstrated sustained stability of the solid tumor components of the primary cavitary lesion and the irradiated metastatic nodule (**Figure 2**). Despite the complexity of quantifying the cavitary lesion via RECIST 1.1, the patient derived clear clinical benefit with a progression-free survival (PFS) of approximately 11 months. A diagnostic challenge occurred in June 2025 when CT showed increased consolidation around the primary lesion. A comprehensive workup—including negative GeneXpert MTB/RIF and repeated sputum cultures—ruled out TB reactivation. The finding was attributed to peritumoral inflammation/obstructive pneumonitis rather than progression or infection. Regarding safety, the patient experienced transient Grade 1–2 transaminase and TSH elevations, which were managed without corticosteroids or treatment discontinuation. Crucially, under concurrent anti-TB therapy, no Grade ≥3 immune-related adverse events (irAEs) occurred, and there was no clinical or radiologic evidence of TB dissemination (**Figure 3**).The detailed clinical course, systemic anticancer regimens, and corresponding responses are summarized in **Table 1**.

**Table 1:** Summary of clinical course, systemic anticancer treatments, and responses.

Time period	Clinical phase	Anticancer/local therapy	Concurrent MDR-TB management	Tumor & infection response
Oct–Dec 2022	Initial diagnosis	—	No active TB detected	Stage IVA SqNSCLC diagnosed
May–Nov 2023	1st-line	Pembrolizumab + liposomal paclitaxel + nedaplatin → pembrolizumab maintenance	No active TB detected	SD → PD
Nov 2023–Mar 2024	2nd-line	Docetaxel (AK112-201 control arm)	No active TB detected	PD
Apr–Jun 2024	3rd-line	Ivonescimab (AK112)	Suspected infection; treatment held	Clinical deterioration with fever/infiltrates
Aug–Dec 2024	MDR-TB control	Systemic therapy held initially	All-oral MDR-TB regimen (bedaquiline, linezolid, clofazimine, cycloserine)	Symptoms improved; imaging inflammation regressed; culture conversion (Dec 2024)
Oct–Dec 2024	Bridge/local control	Consolidative IMRT to LUL lesion (66 Gy/33f)	Continued MDR-TB therapy with monitoring	Irradiated lesion shrank
Jan–Feb 2025	Rechallenge (phase 1)	QL1706 monotherapy	Ongoing MDR-TB therapy	SD; no TB reactivation
Feb–May 2025	Rechallenge (phase 2)	QL1706 + short-course chemotherapy	Ongoing MDR-TB therapy	SD; chemo stopped for toxicity
Jun 2025–Nov 2025	Maintenance	QL1706 maintenance	Ongoing MDR-TB therapy	Durable SD (~11 months PFS); sustained TB negativity

## 3 Discussion

To our knowledge, this is among the first reports describing PD-1/CTLA-4 dual checkpoint blockade in an advanced squamous NSCLC patient with controlled MDR-TB on ongoing anti-TB therapy. The patient achieved durable disease stabilization (estimated PFS ~11 months) after rechallenge with QL1706, without microbiologic or radiologic evidence of TB reactivation and without ≥ grade 3 immune-related adverse events.

The coexistence of advanced lung cancer and TB poses a major therapeutic dilemma. Patients with active TB are generally excluded from ICI trials, and clinical reports have raised concerns about TB reactivation or new-onset TB during immune checkpoint blockade (2, 3, 10–13). In the present case, the decision to resume immunotherapy was supported by objective evidence of infection control, including clinical improvement, regression of inflammatory infiltrates, and subsequent sustained microbiologic negativity during ongoing MDR-TB therapy.

Several pragmatic factors likely contributed to the favorable outcome. First, systemic anticancer therapy was deferred to prioritize infection control, and QL1706 was initiated only after stabilization on a guideline-concordant MDR-TB regimen with intensified surveillance. Second, local consolidative radiotherapy controlled a progressing thoracic lesion and reduced tumor burden, creating a window for systemic rechallenge. Third, QL1706 provides dual PD-1/CTLA-4 blockade with an optimized exposure profile of the anti-CTLA-4 component. While definitive conclusions cannot be drawn from a single case, this feature may have contributed to manageable toxicity and granuloma preservation; however, any mechanistic explanation in the setting of MDR-TB remains hypothesis-generating (4, 5). Mechanistically, the addition of CTLA-4 blockade may recruit new T-cell clones via lymph node priming (9), overcoming the resistance to prior PD-1 and PD-1/VEGF inhibitors.

Pragmatic considerations. Based on this case, we suggest the following when considering ICIs/dual checkpoint blockade in NSCLC patients with controlled MDR-TB: (1) confirm infection control using symptoms, inflammatory markers, imaging, and serial microbiology; (2) select patients with preserved functional status and ability to adhere to prolonged anti-TB therapy; (3) consider local therapy to control dominant progressing lesions; (4) interpret radiologic changes cautiously and differentiate tumor progression from infection and treatment-related inflammation via multidisciplinary evaluation; and (5) intensify monitoring for hepatotoxicity and pulmonary complications given overlapping risks from anti-TB therapy and immunotherapy (10–14).

Limitations. This report has several limitations. The contribution of radiotherapy and short-course chemotherapy cannot be separated from the effect of QL1706. Response assessment was challenged by a cavitary lesion with overlapping inflammatory changes, limiting formal RECIST evaluation. As a single case, these observations are not generalizable and require validation in larger cohorts.

## 4 Conclusion

This case highlights that the coexistence of MDR-TB and advanced NSCLC does not necessarily preclude the use of dual PD-1/CTLA-4 checkpoint blockade. Our experience suggests that QL1706 can be a feasible salvage strategy in this challenging population, provided that infection is first controlled with a guideline-concordant MDR-TB regimen and that treatment is guided by a rigorous multidisciplinary team. While strict surveillance is mandatory to prevent reactivation, this report offers preliminary evidence that dual immunotherapy may be safely administered to carefully selected patients with chronic tuberculosis, potentially expanding treatment access for this neglected demographic.
