# Correction: Case Report: PD-1/CTLA-4 dual checkpoint blockade(QL1706) in advanced pulmonary squamous cell carcinoma complicated by multidrug-resistant tuberculosis after multiple lines of immunotherapy

**DOI:** 10.3389/fonc.2026.1896026

**Published:** 2026-06-11

**Authors:** Li Huang, Hao Li

**Affiliations:** 1 Department of Oncology, Guiyang Public Health Clinical Center, Guiyang, China; 2 Department of Oncology, Guiyang Cancer Hospital, Guiyang, China; 3 Department of Oncology, Guiyang Fifth People’s Hospital, Guiyang, Guizhou, China

**Keywords:** immunotherapy rechallenge, multidrug-resistant tuberculosis, non-small cell lung cancer, PD-1/CTLA-4, QL1706, squamous cell carcinoma

The article type of this published article was erroneously assigned as “Review”. The correct article type is “Case Report”.

The original version of this article has been updated.

